# An Update on Antiviral Therapy Against SARS-CoV-2: How Far Have We Come?

**DOI:** 10.3389/fphar.2021.632677

**Published:** 2021-03-08

**Authors:** Omkar Indari, Shweta Jakhmola, Elangovan Manivannan, Hem Chandra Jha

**Affiliations:** ^1^ Department of Biosciences and Biomedical Engineering, Indian Institute of Technology Indore, Indore, India; ^2^ School of Pharmacy, Devi Ahilya Vishwavidyalaya, Indore, India

**Keywords:** COVID-19, SARS-CoV-2, drug repurposing, antivirals, mechanism of action, pharmacokinetics

## Abstract

COVID-19 pandemic has spread worldwide at an exponential rate affecting millions of people instantaneously. Currently, various drugs are under investigation to treat an enormously increasing number of COVID-19 patients. This dreadful situation clearly demands an efficient strategy to quickly identify drugs for the successful treatment of COVID-19. Hence, drug repurposing is an effective approach for the rapid discovery of frontline arsenals to fight against COVID-19. Successful application of this approach has resulted in the repurposing of some clinically approved drugs as potential anti-SARS-CoV-2 candidates. Several of these drugs are either antimalarials, antivirals, antibiotics or corticosteroids and they have been repurposed based on their potential to negate virus or reduce lung inflammation. Large numbers of clinical trials have been registered to evaluate the effectiveness and clinical safety of these drugs. Till date, a few clinical studies are complete and the results are primary. WHO also conducted an international, multi-country, open-label, randomized trials-a solidarity trial for four antiviral drugs. However, solidarity trials have few limitations like no placebos were used, additionally any drug may show effectiveness for a particular population in a region which may get neglected in solidarity trial analysis. The ongoing randomized clinical trials can provide reliable long-term follow-up results that will establish both clinical safety and clinical efficacy of these drugs with respect to different regions, populations and may aid up to worldwide COVID-19 treatment research. This review presents a comprehensive update on majorly repurposed drugs namely chloroquine, hydroxychloroquine, remdesivir, lopinavir-ritonavir, favipiravir, ribavirin, azithromycin, umifenovir, oseltamivir as well as convalescent plasma therapy used against SARS-CoV-2. The review also summarizes the data recorded on the mechanism of anti-SARS-CoV-2 activity of these repurposed drugs along with the preclinical and clinical findings, therapeutic regimens, pharmacokinetics, and drug-drug interactions.

## Introduction

**FIGURE 1:**
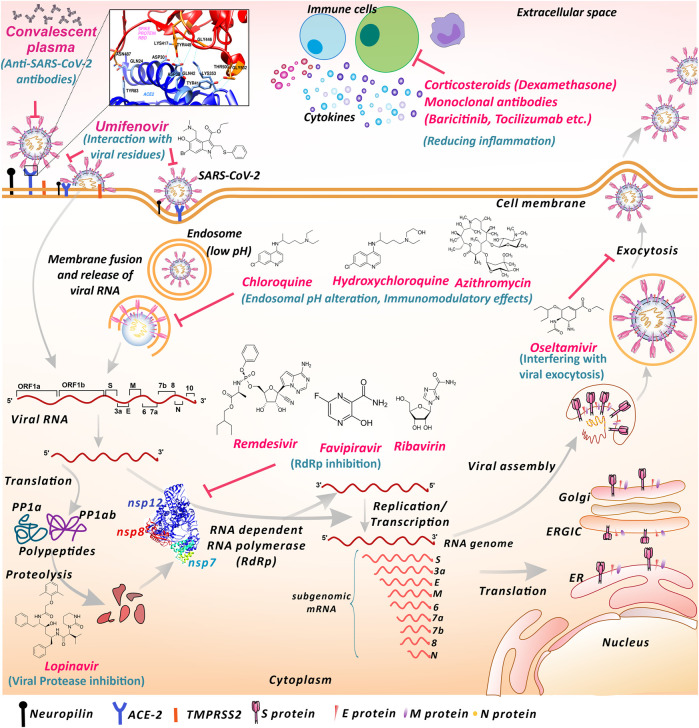
Proposed mechanisms of repurposed drugs and therapies used against SARS-CoV-2 infection. SARS-CoV-2 interacts with cell surface receptors like ACE-2 and neuropilin to gain entry inside the cell. Umifenovir may interact with SARS-CoV-2 surface glycoproteins and lipids and obstruct the interaction with the entry receptor ACE-2. Anti-SARS-CoV-2 antibodies present in convalescent plasma may inhibit SARS-CoV-2 entry and subsequent infection transmission. Chloroquine, hydroxychloroquine and azithromycin may elevate endosomal pH and hinder viral entry and RNA release process. Chloroquine, hydroxychloroquine and azithromycin also shows immunomodulatory effects. Nucleoside inhibitors like remdesivir, favipiravir and ribavirin may inhibit RNA replication and suppress RNA-dependent RNA polymerase activity. Lopinavir may fraternize with viral protease altering the proteolysis. Oseltamivir may interplay with components involved in the exocytosis process, blocking the viral exit from the cell. Monoclonal antibodies against cytokine receptors and Corticosteroid shows anti-inflammatory actions against exaggerated immune response. (ACE-2-Angiotensin-converting enzyme 2, TMPRSS2 Transmembrane Serine protease 2, RdRp- RNA dependent RNA polymerase, ER- Endoplasmic reticulum, ERGIC- Endoplasmic reticulum-golgi intermediate complex. The displayed ACE-2-Spike interaction residues and RdRp structures are based on Protein databank structure ID: 6M0J and 6M71 respectively).

The coronavirus disease of 2019 (COVID-19) pandemic has changed the scenario of the entire world, which has not been seen for a century. Influenza (H1N1 virus) outbreak of Spain that occurred in 1918 was the worst ever hit pandemic in recent history. Now, the current outbreak started in Wuhan, China, has globally spread to 219 countries and territories (WHO, 2020a). Severe acute respiratory syndrome coronavirus (CoV) 2 (SARS-CoV-2), a large ssRNA virus, is the causative agent of COVID-19, which primarily attacks the respiratory tract including associated organs. Additionally, the virus has shown to impact various other organs or body systems like the gastrointestinal system, nervous system etc (Jakhmola et al., 2020a; Jakhmola et al., 2020b; Sonkar et al., 2020). Currently new variants of SARS-CoV-2 are reported from different regions of the world. In December 2020, the United Kingdom variant of SARS-CoV-2 lineage B.1.1.7, now designated as Variant of Concern 202012/01 (VOC) and the South Africa variant named 501Y.V2 have been reported to spread widely within the country and displaced the other lineages of viruses (WHO, 2020c). By the end of first COVID-19 pandemic year the VOC-202012/01 variant was reported in 31 other countries/territories (WHO, 2020c). The receptor-binding domain of viral spike protein is essential in SARS-CoV-2 entry into the host cell via surface angiotensin-converting enzyme-2 (ACE-2) (Zhou et al., 2020) (Figure 1). Recently, another cell receptor Neuropilin-1 was found to be involved in SARS-CoV-2 entry (Cantuti-Castelvetri et al., 2020). The further life cycle of the virus inside the cell is similar to that of other coronaviruses. After binding to the receptor, the conformational change in the spike protein leads to virus fusion with the host cell membrane. The virus may transfer the RNA directly inside the cells or may proceed through the endosomal pathway (Simmons et al., 2005; Li, 2016; Hasan et al., 2020; Hoffmann et al., 2020). Upon translation of viral RNA, the viral replicase polyprotein PP1a and PP1ab are synthesized and cleaved into small products by viral endopeptidase (Van-Boheemen et al., 2012; Shereen et al., 2020). RNA dependent RNA polymerase (RdRp) produces subgenomic RNAs by discontinuous transcription (Hussain et al., 2005; Chen et al., 2020; Shereen et al., 2020). This further gets translated into respective viral proteins. After processing through the endoplasmic reticulum (ER), ER-Golgi intermediate compartment (ERGIC), and Golgi complex the viral RNA and proteins are assembled into virions (Lai and Cavanagh, 1997; Song et al., 2004). These virions are transported through vesicles and exocytosed for transmission. These steps of the viral life cycle are lucrative virus inhibition targets for different drugs (Figure 1).

Currently, no specific drug or vaccine is available for the treatment of SARS-CoV-2 infected patients. Nonetheless, drug repurposing could prove to be advantageous tactics for finding COVID-19 treatment. Benefits of drug repurposing include cost-effectiveness, elimination of some clinical trial steps, sooner on-field availability, combining the candidate drugs with other possible drugs based on prior data, and generation of new information about the existing drugs mechanisms (Agrawal, 2015). The available knowledge of previous CoVs treatments, genomic sequences, and protein modeling studies has helped researchers put forward the potential COVID-19 drug candidates. The primarily investigated drugs are either antimalarials, antivirals, antibiotics, corticosteroids, and they have been repurposed based on their potential either to negate virus, reduce lung inflammation or other disease symptoms. In particular, chloroquine (CQ) hydroxychloroquine (HCQ) and azithromycin (AZM) are majorly used against COVID-19 as they initially showed reasonably good *in vitro* and *in vivo* antiviral activity against SARS-CoV, MERS-CoV and SARS-CoV-2. Lopinavir/ritonavir (LPV/RTV), which are anti-HIV drugs, were examined for COVID-19 as they were found to be effective in earlier CoV outbreaks. Moreover, remdesivir (RDV), an experimental anti-Ebola drug, was investigated for COVID-19 and received greater attention. Based on the appreciable preliminary data, FDA has issued EUA for CQ, HCQ, and RDV (FDA, 2020b; FDA, 2020c; FDA, 2020d). However, later the EUA for CQ and HCQ was revoked while EUA for RDV was re-issued by some amendments. Similarly, favipiravir (FPV), ribavirin (RBV), umifenovir (UFV), and oseltamivir (OTV) having a broad-spectrum antiviral activity were also clinically investigated against SARS-CoV-2. WHO put forward a solidarity clinical trial, a multi-country, open-label randomized trial, for the use of HCQ, RDV, LPV/RTV, or LPV/RTV in combination with Interferon (IFN) β-1a against COVID-19 (WHO, 2020b). The recent interim results of the solidarity trial declare that all these drugs had little or no effect on overall mortality, initiation of ventilation and duration of hospital stay in hospitalized patients (Pan et al., 2020). So far, to treat severe and critical COVID-19, only corticosteroids have proven effective (WHO, 2020b). New treatment options need to be added to the solidarity trial in the future. However, solidarity trials may have few limitations like it was focused on worldwide outcomes of considered treatments. Hence, if a particular drug shows comparatively better outcome in some regions or populations that could get neglected. Therefore, there is scope to conduct the clinical studies involving various suitable drugs with different treatment regimens and combinations.

In this review, we have provided updated comprehensive information about majorly repurposed drugs which are used as antivirals to combat SARS-CoV-2 infection**.** We attempted to collate and review the studies regarding all these drugs, which are dispersed in various distinct publications. Furthermore, we have also summarized the data recorded on the mechanism of anti-SARS-CoV-2 activity of these repurposed drugs along with the preclinical and clinical findings, therapeutic regimens, pharmacokinetics, and drug-drug interactions.

## Selected Antivirals Repurposing in COVID-19 Treatments

### Chloroquine and Hydroxychloroquine

CQ and HCQ both belong to the 4-aminoquinoline chemical class (Devaux et al., 2020) with potential antimalarial and anti-inflammatory activities. These drugs are weak diprotic bases that increase the endosomal pH to hinder the host-virus fusion process (Devaux et al., 2020) (Figure 1; Table 1). *In vitro* studies have shown antiviral activity of CQ on MERS and SARS-CoV (Cong et al., 2018; Keyaerts et al., 2004). In addition, *in vivo* studies suggest potent activity of these drugs against human CoV-OC43, EV-A71, zika virus, and *in vitro* activity against influenza-A (Keyaerts et al., 2009; Tan et al., 2018; Li et al., 2017; Ooi et al., 2006). Recent *in vitro* studies report CQ and HCQ effectiveness against SARS-CoV-2 (Half maximal effective concentration (EC50) 2.71mM and 4.51mM, respectively) in Vero E6 cells (Liu J. et al., 2020). However, HCQ has *in vitro* activity with a lower EC50 for SARS-CoV-2 compared to CQ after 24h of growth (HCQ: 6.14μM and CQ: 23.90μM) (Yao X. et al., 2020). CQ treatment has demonstrated to reduce the recovery time and improved physiological conditions in COVID-19 patients. According to a randomized Chinese COVID-19 controlled trial, CQ (Dose 500mg bid, 15days) may work more efficiently than LPV/RTV (Huang M. et al., 2020). Another study compared the low dose (450mg bid for 1day followed by 450mg, 4days) and high dose (600mg bid, 10days) in combination with azithromycin (AZM) and OTV which determined that high dose CQ was associated with high mortality (Borba et al., 2020). A multicentre, randomized, open-label trial from China investigated the use of HCQ (1200mg daily for 3days, followed by a maintenance dose of 800mg daily) to standard care. The interpretation included that the HCQ treated group showed inadequate response compared to control (Tang et al., 2020). The combination of HCQ and AZM resulted in early viral clearance, as demonstrated by an open-label non-randomized clinical trial (Gautret et al., 2020). A meta-analysis report stated that compared to alone HCQ, the combination of HCQ and AZM significantly increased mortality in COVID patients (Fiolet et al., 2020). A United States based observational study interpreted that HCQ treated patients did not either benefit or suffer in terms of intubation or mortality (Geleris et al., 2020). A large-scale clinical trial was conducted in United Kingdom, a Randomized Evaluation of COVID-19 Therapy (RECOVERY Trial), to investigate various drug candidates or therapies including HCQ against severe COVID-19. The result demonstrated no efficacy of HCQ against COVID-19 (Horby et al., 2020b). Surprisingly FDA issued EUA for CQ and HCQ against COVID-19 on March 28, 2020 and was revoked on June 15, 2020 (FDA, 2020b; FDA, 2020c). Major side effects of these drugs include QT prolongations, and decreased insulin clearance and resistance (FDA, 2020b; FDA, 2020c). The overuse of CQ and HCQ could possibly lead to tissue injury in the liver, retina, skeletal, and cardiac muscle cells due to their lysosomal affinity (Satarker et al., 2020; Cohen, 2020). Therefore, studies recommend that physicians avoid high doses and exercise extreme caution in the compassionate use of CQ/HCQ, either alone or in combination with other antivirals (Acharya and Sayed, 2020). Currently 88 and 267 COVID-19 associated clinical trials have been registered for CQ and HCQ respectively (ClinicalTrials.gov, 2020b; ClinicalTrials.gov, 2020d).

**TABLE 1:** General information of repurposed drugs used against SARS-CoV-2.

Drugs	Group		Mechanism of action	Targeted virus/disease indication	Molecular target	Possible correlation to be used against COVID 19 treatment	No. of clinical trials registered	Strengths	Limitations
CQ or HCQ		Antiparasitic	Antiviral effect, immuno-modulation	*Plasmodium* sp., arthritis, CoV-OC43, enterovirus 71, zika virus	Altering endosomal pH	Activity against SARS-CoV2 and immuno-modulatory effect	88 and 267	Have shown activity against earlier outbreak CoVs	Shown to cause QTc prolongation, torsades de pointes, ventricular arrhythmia, and cardiac deaths
LPV/RTV		HIV protease inhibitor	Viral protease inhibition	HIV, SARS-CoV, MERS-CoV	Viral protease inhibition	Binding to M^pro^ protein of SARS-CoV-2	90	Have shown activity against earlier outbreak CoVs	No efficacy in multiple clinical trials including large scale clinical trials, known to cause QTc prolongation and is on the possible risk of torsades de pointes
RDV		Nucleoside analogue	Viral RNA synthesis termination	Ebola, SARS-CoV, MERS-CoV, yellow fever virus, dengue virus type 2, influenza A, parainfluenza 3, and various delta CoVs	Adenosine analogue competes with dATP in RNA synthesis	Viral replication inhibition	78	Recently discovered drug active against multiple viruses including delta CoVs, SARS and MERS CoVs, shown efficacy in recent clinical trials	Questionable safety on long term effect as the drug is recently discovered, showed no efficacy in large scale trials, known to cause acute hepatotoxic effect due to an increase in hepatic transaminase activity but no effect on QTc
FPV		Nucleoside analogue	Viral RNA synthesis inhibition	Influenza a and B viruses, arenavirus, bunyavirus, flavivirus, filoviruses and ebola virus	Guanosine analogue competes with dGTP in RNA synthesis	Viral replication inhibition	45	Active against many viruses, shown *in vitro* activity against SARS-CoV-2	Variation in FPV plasma concentration between the US and the Japanese population, shown to cause adverse effects on the fetus
RBV		Nucleoside analogue	Viral RNA synthesis inhibition	Hepatitis C virus, canine distemper virus, enterovirus 71, chikungunya virus, semliki forest virus, orthopoxvirus, influenza virus, flavi- and paramyxoviruses	Guanosine analogue competes with dGTP in RNA synthesis	Viral replication inhibition	15	Shows efficacy against MERS-CoV in animal model and used in earlier CoV outbreaks	Majorly used in combination with other drugs and is not effective against reducing mortality, shown to cause hemolytic anemia and worsening of cardiac disease to myocardial infarctions
AZM		Antibiotic	Bacterial protein synthesis inhibition, Antiviral effect	Bacteria, influenza virus, dengue virus, zika virus, ebola virus	Altering endosomal pH	Activity against SARS-CoV2, immuno-modulatory effect and interfere with viral replication	122	Active against many viruses and shown *in vitro* activity against SARS-CoV-2	Majorly used in combination with other drugs, showed adverse events, no efficacy in large scale trials, shown to cause QTc prolongation including ventricular and supraventricular arrhythmia
UFV		Broad spectrum antiviral	Stacking interactions with certain amino acid residues, viral glycoproteins, lipids	Influenza-A virus, respiratory syncytial virus, rhinovirus type-14, Coxsackievirus-B3 and adenovirus type-7	Stacking interactions with certain amino acid residues, viral glycoproteins, lipids	Targeting viral proteins or lipids and preventing viral entry	11	Active against SARS-CoV and SARS-CoV-2 *in vitro*, commonly used	No efficacy against COVID-19, rarely cause serious mental/mood changes but no effect on QTc
OTV		Neuraminidase inhibitor	Inhibits viral neuraminidase enzyme	Influenza a and B viruses	Component involved in exocytosis process	Virus exocytosis Inhibition	20	Commonly used drug	No efficacy against SARS-CoV-2, rarely cause serious mental/mood changes but no effect on QTc

The strength and limitations of drug used are conclusively stated comparing the reports explained in the manuscript. QTc: corrected QT interval.

### Lopinavir/Ritonavir

LPV/RTV are approved anti-HIV drugs that specifically target HIV protease (Chandwani and Shuter, 2008). LPV is used in combination with RTV to elevate its half-life via cytochrome P450 suppression (Chandwani and Shuter, 2008). LPV is predicted to act on the viral 3-chymotrypsin-like protease (3CLpro) (Sisay, 2020) (Figure 1; Table 1). Previous studies have established *in vitro* LPV effectiveness against SARS-CoV at 4μg/ml (Chu et al., 2004). A cumulative *in vivo* study involving LPV and RTV against MERS revealed the EC50 of the two drugs as 11.6 and 24.9µM respectively with 50% cytotoxic concentration (CC50) values >50µM (Sheahan et al., 2020). However, no *in vitro* study is available of LPV/RTV against SARS-CoV-2. In China, a clinical trial for LPV/RTV against adult hospitalized COVID-19 patients was conducted (Cao et al., 2020). The study showed no benefit of LPV/RTV (Dose: 400mg/100mg bid, 14days) treatment compared to standard care control groups (Cao et al., 2020). Although LPV/RTV treated groups exhibited less serious complications than the controls. A Japanese case-study reports successful treatment of non-severe COVID-19 pneumonia patients with LPV/RTV (Wada et al., 2020). Another study of 47 patients reported that LPV/RTV treatment improved physiological condition without adverse events (Ye et al., 2020). In the study of 120 patients, if the LPV/RTV treatment is initiated within 10days of symptom onset, it significantly reduces viral shedding (Yan et al., 2020). LPV/RTV along with IFN-⍺ or RBV may improve the health of COVID-19 patients (Yuan et al., 2020). On the contrary, another report from Taiwan suggested that LPV/RTV treatment did not reduce the duration of viral shedding in infected patients (Cheng et al., 2020). A single-center, randomized, open-labeled, prospective clinical trial conducted by Huang *et al.* studied the effect of LPV/RTV plus IFN-α, LPV/RTV plus RBV plus IFN-α, and RBV plus IFN-α treatment on COVID-19 patients. All the three regimens showed no significant difference regarding their effectiveness against COVID-19. LPV/RTV when given in combination with RBV lead to more adverse events suggesting that these two drugs should not be administered together (Huang Y.-Q. et al., 2020). A meta-analysis study investigating randomized trials showed LPV/RTV may reduce mortality (Huang Y.-Q. et al., 2020; Verdugo-Paiva et al., 2020). Albeit some contradictory studies showed no statistically significant effect on reducing the death rate (Karolyi et al., 2020; Horby et al., 2020). A report stated the risk of bradycardia in elderly critically ill COVID-19 patients with RTV plasma overdose (Beyls et al., 2020). Adverse gastrointestinal effects such as diarrhea, nausea and vomiting have been observed in patients treated with LPV/RTV (Huang Y.-Q. et al., 2020; Liu W. et al., 2020; Vecchio et al., 2020). Therefore, it remains difficult to safely recommend LPV/RTV dose without compromising the benefit of the antiviral strategy. There is an urgency of a comprehensive pharmacokinetic/pharmacodynamic analysis for the upcoming clinical trials in similar critically ill COVID-19 patients (Lê et al., 2020). Currently, 90 clinical trials have been registered for LPV/RTV for COVID-19 (ClinicalTrials.gov, 2020e).

### Remdesivir

RDV is a prodrug of an adenosine triphosphate (ATP) analog and is converted into its active form GS-441524 on administration (Al-Tawfiq et al., 2020). It was initially proposed for the Ebola virus that acted by viral replication inhibition through premature termination of RNA transcription (Al-Tawfiq et al., 2020) and hence targeting RdRp. RDV metabolites have proved useful against yellow fever virus, Dengue virus type 2, influenza A, parainfluenza 3, and various delta CoVs (Cho et al., 2012). The parent nuclei of RDV is evaluated against alpha CoV (EC50 = 0.78μM), porcine delta CoV, SARS-like bat CoVs and MERS-like bat CoVs (Murphy et al., 2018; Brown et al., 2019; Amirian and Levy, 2020). Studies on SARS and MERS infected human airway epithelial cells (EC50 ≈ 0.07μM) and animal models demonstrated the viral polymerase inhibition ability of the drug (Agostini et al., 2018). The drug is efficient at EC50, 0.77μM, and CC50 > 100μM in Vero E6 cells against SARS-CoV-2 (Wang M. et al., 2020). Studies also revealed that RDV targeted structurally analogous regions of SARS-CoV-2 polymerase (Lo et al., 2020). Several *in-vivo* reports suggested that RDV decreased viral load, reduced pathological processes, alleviated mild symptoms, and improved pulmonary lesions in SARS-CoV-2 infected animals with adverse effect (Frediansyah et al., 2020; Badgujar et al., 2020; Pruijssers et al., 2020). The recommended dose of RDV is 200mg on Day 1 and 100mg daily for 5days (for non-severe cases) to 10days (severe cases). A similar dose was considered in numerous clinical trials. A randomized, open-label, phase 3 trial investigating RDV dose for 5days vs. 10days revealed that the treatment for 5days was comparatively beneficial (Spinner et al., 2020). A double-blinded, randomized, placebo-controlled trial, determined that severe COVID-19 patients treated with RDV showed fast recovery compared to control, though statistically insignificant (Wang Y. et al., 2020). Moreover, the RDV administration is not approved globally due to questionable safety. Although SOLIDARITY trial results denote that RDV is not beneficial against COVID-19, result of some recently completed clinical trials are contrary. A double-blinded, randomized, placebo-controlled trial from the United States showed that RDV treated hospitalized patients may recover faster with comparatively less adverse events and mortality than the placebo group (Beigel et al., 2020). Prominent adverse reactions were acute respiratory failure, decreased glomerular filtration rate, lymphocytopenia, pyrexia, hyperglycemia, increased anemia, increased creatine, and liver transaminases (Beigel et al., 2020; FDA, 2020d). Result of multicentre clinical trial published at the end of first year of the pandemic, showed that RDV given in combination with baricitinib (a Janus kinase inhibitor used to hinder intracellular signaling of cytokines) was effective compared to RDV alone in terms of reducing recovery time additionally speeding improvement (Kalil et al., 2020). Based on such positive results of RDV, it has been approved to use by various authorized platforms like FDA (Mahase and McCullough, 2020). An interesting investigation showed that RDV’s parent nucleotide GS-441524 is superior and less toxic than its pro-drug form and has shown efficacy in *in vivo* veterinary settings (Yan and Muller, 2020). Therefore, further investigation regarding the use of the parent nucleotide itself against COVID-19 should be driven with a faster pace. Currently, 78 COVID-19 associated clinical trials are registered with RDV (ClinicalTrials.gov, 2020g).

### Favipiravir

Favipiravir (FPV), an approved influenza treatment, is a pyrazinecarboxamide derivative (Furuta et al., 2013). It also showed efficacy against arenavirus, bunyavirus, flavivirus, filoviruses, and Ebola virus (Furuta et al., 2017). The prodrug after administration is transformed by host enzymes into the ribofuranosyl triphosphate derivative (T-705-RTP), a guanine analogue and suppresses the RdRp (Figure 1; Table 1). *In vitro* effectivity of FPV against SARS or MERS viruses have not been addressed. An *in vitro* study has shown inhibition of SARS-CoV-2 by FPV (EC50 = 61.88μM; CC50 = over 400μM) (Wang X. et al., 2020). In Japan, the approved dose of FPV against influenza is 1,600mg bid on day 1, followed by 600mg bid on days 2–5 with associated side effects (PMDA, 2020). A Chinese open-label, controlled study investigated the effects of FPV (Day 1; 1600mg twice and Day 2–14; 600mg bid) vs. LPV/RTV (Day 1–14; 400mg/100mg bid). The preliminary results indicated potent FPV action and fewer adverse effects than LPV/RTV (*p* < 0.001) (Cai et al., 2020). A report suggested treatment of COVID-19 patients with FPV during times of early symptoms, helped in reducing the SARS-CoV-2 presence in nasal secretions (McCullough, 2020). However, previous clinical trials have reported the variation in FPV plasma concentration between the United States and the Japanese population (Madelain et al., 2016). Therefore, more trials regarding global use of FPV should be considered. In a Japanese study FPV also showed to control inflammatory mediators and pneumonia progression in COVID-19 patients (Yamamura et al., 2020). Severe or critical COVID-19 patients showed improvements after treating with FPV (Takahashi et al., 2020) and FPV also led to improved lung histology (Kaptein et al., 2020). However, in a meta-analysis study, FVP proved to have significant clinical and radiological improvement without significant differences on viral clearance (Shrestha et al., 2020). For the use of FPV with respect to COVID-19, 45 clinical trials have been registered (ClinicalTrials.gov, 2020c).

### Ribavirin

Ribavirin (RBV), a broad-spectrum antiviral prodrug is metabolized in host into a guanosine analog (Gish, 2006). The drug showed antiviral efficacy against canine distemper virus, hepatitis C virus, Enterovirus 71, Chikungunya virus, and Semliki Forest virus, orthopoxvirus, influenza virus, flavi- and paramyxoviruses (Elia et al., 2008; Galli et al., 2018; Li et al., 2008; Briolant et al., 2004; Smee et al., 2001; Leyssen et al., 2005). A study observed reduced replication of the MERS-CoV in rhesus macaques upon treatment with IFN-α2b and RBV (Falzarano et al., 2013). RBV in combination with LPV/RTV was used in SARS-CoV and MERS-CoV trials (Yao T. et al., 2020). In the case of SARS-CoV-2 infection, an *in vitro* study determined the EC50 of RBV as 109.50uM (Wang X. et al., 2020). A study included RBV along with LPV/RTV and IFN-α in the treatment of hospitalized COVID-19 patients (Hung et al., 2020). The triple therapy was found to be beneficial to reduce disease symptoms and virus shedding compared to groups provided LPV-RTV alone. The dose of RBV considered was 400mg bid along with 400mg/100mg of LPV/RTV + IFN-α for 14days. A study assessed the impact of sofosbuvir/daclatasvir (antivirals) compared to RBV in treatment of COVID-19 patients. The mortality was higher (33%) in COVID-19 patients treated with RBV than that of sofosbuvir/daclatasvir (Eslami et al., 2020). A retrospective cohort study comparing RBV vs. supportive therapy stated that RBV did not help in reducing the mortality rate in COVID-19 patients (Tong et al., 2020). 15 clinical trials have been registered for the use of RBV alone or in combination with other COVID-19 drugs (ClinicalTrials.gov, 2020h).

### Azithromycin

Azithromycin **(**AZM) is a semisynthetic macrolide antibiotic belonging to the azalide class (Ballow and Amsden, 1992). It has bactericidal effects and targets the protein synthesis process of bacteria. AZM has also been shown to inhibit influenza, zika, dengue, and Ebola viruses (Damle et al., 2020; Wang M. et al., 2020). Specifically, a study showed AZM induced reduction in rhinovirus replication 7-fold in primary bronchial epithelial cells without inducing cell death (Schögler et al., 2015). The *in vitro* EC50 for AZM against SARS‐CoV‐2 was 2.12µM (EC90: 8.65µM) following a 72‐hour incubation post-infection (MOI of 0.002) (Hughes et al., 2020). The addition of AZM with HCQ was efficient in virus elimination in COVID-19 patients (Gautret et al., 2020). The dose of 500mg on day 1 followed by 250mg/day, the next 4days was used in compliment to HCQ dose of 200mg, three times/day, for 10days. Some investigations suggested HCQ and AZM combination to be beneficial in reducing mortality in COVID-19 patients (Bonny et al., 2020; Arshad et al., 2020). A case report showed AZM provided with HCQ proved to be an effective treatment approach in pregnant women against the SARS-CoV-2 infection and associated with reduced mortality (Sisti et al., 2020). In contrast, a report from the United States stated that neither HCQ nor AZM separately or together could reduce the mortality of COVID-19 patients compared to the control group (Rosenberg et al., 2020). Moreover, treatment of AZM and HCQ was associated with greater changes in QTc in COVID-19 patients (Mercuro et al., 2020). Few other studies also reported that AZM included in treating COVID-19 patients did not provide any beneficial effect (Rodríguez-Molinero et al., 2020; Furtado et al., 2020; Cavalcanti et al., 2020). 122 clinical trials have been registered for the use of AZM alone or in combination with other drugs against COVID-19 (ClinicalTrials.gov, 2020a).

### Umifenovir

Umifenovir (UFV) is an indolyl carboxylic acid widely recognized as Arbidol (Blaising et al., 2014). It is used as a treatment and prevention measure against influenza virus (Blaising et al., 2014). It has direct antiviral and host-targeting action. UFV can interact with virus protein or lipid components and may hinder different stages of the viral life cycle (Blaising et al., 2014). *In vitro* analysis of the antiviral activity of arbidol against several human respiratory viruses, namely influenza-A virus, respiratory syncytial virus, rhinovirus type-14, coxsackievirus-B3 and adenovirus type-7 is demonstrated (Shi et al., 2007). Inhibition of SARS-CoV replication on UFV treatment was demonstrated *in vitro*. UFV is also known to inhibit various isolates of zika virus in multiple cell lines (Fink et al., 2018). The inhibitory action of the drug against SARS-CoV-2 in Vero E6 cells (MOI of 0.05) has been demonstrated. The EC50 and CC50 were 4.11 and 31.79μM, respectively (Wang X. et al., 2020). Briefly, the study showed enhanced inhibitory activity at early stages compared to the post-entry stage (Figure 1). A small-scale study suggested post-exposure prophylaxis (PEP) use of UFV in people exposed to COVID-19 patients (Zhang et al., 2020). Another study determined that arbidol monotherapy was superior to LPV/RTV against COVID-19 (Zhu et al., 2020). COVID-19 patients provided with UFV along with LPV/RTV showed better outcomes compared to patients who received LPV/RTV only (Deng et al., 2020). A contrary study reported that UFV was not beneficial to improve the condition of the patient or viral clearance (Lian et al., 2020). Moreover, another study suggested arbidol + LPV/RTV were associated with many adverse events (Wen et al., 2020). In most of the studies, a dose of 200mg thrice a day was considered. According to a meta-analysis, UFV was not effective in terms of reducing the SARS-CoV-2 elimination from the infected patient in terms of detection in diagnostic tests and even hospital length of stay of hospitalized patients (Huang D. et al., 2020). There is no evidence to support the use of UFV for improving patient‐important outcomes in patients with COVID‐19. 11 registered clinical trials include UFV use in COVID-19 treatment (ClinicalTrials.gov, 2020i).

### Oseltamivir

Oseltamivir (OTV) is a synthetic derivative prodrug of ethyl ester with antiviral activity (Schade et al., 2014). It acts as a neuraminidase inhibitor against the influenza virus and is also effective for various avian influenza virus strains (Ward et al., 2005). An *in vitro* OTV study on H5N1 influenza showed that the IC50 was 0.1–4.9nM (Govorkova et al., 2009). However, an *in vivo* study involving H5N1 infection required a longer course and higher dosage of OTV (Borio et al., 2018). No *in vitro* study against SARS-CoV-2 is conducted for OTV. The COVID-19 originated in China during flu season, and hence earlier, many patients received OTV treatment until the causative agent SARS-CoV-2 was discovered. Some current clinical trials have used OTV in combination with other major therapeutic candidates. A study showed that the drug exhibited no positive result on COVID-19 (Wang D. et al., 2020). 20 clinical trials have been registered which include OTV in the treatment panel of COVID-19 (ClinicalTrials.gov, 2020f).

## Other Potential Antiviral Drugs and Therapies

### Convalescent Plasma Therapy

Apart from antiviral drugs another probable efficient antiviral strategy includes the use of convalescent plasma collected from the recovered patients containing the anti-SARS-CoV-2 antibodies. Convalescent plasma obtained from patients recovered from COVID-19 carry receptor binding domain specific antibodies with potent antiviral activity (Robbiani et al., 2020). These antibodies can directly interact with SARS-CoV-2 proteins and could block the viral entry into the cell (Figure 1). In August 2020, FDA issued EUA for the use of convalescent plasma in hospitalized patients (FDA, 2021a). The samples were considered of high titer if it followed one of the following criteria-a neutralizing antibody titer of ≥250 as per Broad Institute’s neutralizing antibody assay, a signal-to-cut off (S/C) of ≥12 as per Ortho VITROS IgG assay, or a level of ≥1:2,880 in the Mount Sinai COVID-19 ELISA IgG Antibody Test (FDA, 2021a; FDA, 2021b; FDA, 2021c). Units with low titer should be specified and considered to use if high titer samples were not available. The initial dose of 200ml is recommended and further the dose is advised as per condition and requirement of the patient. However, clinical trials have used different values of titer or doses and generally convalescent plasma was examined using immunoassays instead of viral neutralization assays. For example, a study reported use of no minimum neutralizing-antibody titer and single dose of 200–500ml plasma as per the patient’s condition (Joyner et al., 2020a). While in an open label phase II multicentre randomized controlled trial (PLACID Trial) from India, two doses of 200ml with titers ranging from 1:20 to ≥1:1,280 (from immunoassay) was used. In a Chinese trial, single dose of median volume of 200–250ml with titer ≥1: 1:640 was used (Li et al., 2020). Although various studies have shown efficacy of this therapy (Ahn et al., 2020; Duan et al., 2020; Abolghasemi et al., 2020; Hegerova et al., 2020; Xia et al., 2020), some clinical trials have demonstrated that use of convalescent plasma did not reduced the hospitalization duration, severity, or mortality compared to the control groups (Simonovich et al., 2020; Li et al., 2020; Agarwal et al., 2020). Recently completed randomized, double-blind, placebo-controlled trial from Argentina showed reduced disease progression in patients treated with high titer (>1:1,000) convalescent plasma (Libster et al., 2021). Also, another multicentre study from Poland stated that convalescent plasma can be given as supportive therapy to COVID-19 patients due to availability and low frequency adverse events (Moniuszko-Malinowska et al., 2020). Another large-scale observational analysis of patients from the United States who received the convalescent plasma put forward the opinion that this therapy could be beneficial if provided in early days of symptoms onset (Joyner et al., 2020b, Effect of Convalescent Plasma on Mortality among Hospitalized Patients with COVID-19: Initial Three-Month Experience, 2020). The titers of neutralizing antibodies from donor and viral titers in recipient should be considered for providing the convalescent plasma and further clinical outcomes should be studied for optimizing the therapy. There is a lack of studies exclusively investigating the effect of convalescent plasma treatment on SARS-CoV-2 infected children or pregnant women. Additionally, the effectivity of convalescent plasma in patients infected with new SARS-CoV-2 variants also needs to be tested. The ongoing trials may shed more light on efficacy of this therapy against COVID-19 patients. However, many trials were terminated due to reduced cases in the study region. Currently, overall 172 clinical trials have been registered to investigate the use of convalescent plasma in COVID-19 patients (ClinicalTrials.gov, 2021a).

### New Antiviral Candidates and Other Potential Therapies On-Board

Other than the repurposed drugs the development of anti-SARS-CoV-2 drugs has been accelerated. Recently, a hydroxymethylketone derivative PF-00835,231 showed potency to block protease of SARS-CoV-2 in pre-clinical experiments (Hoffman et al., 2020). This drug has also shown to have suitable pharmaceutical properties and has gathered as an intravenous therapy to cure the disease. Another drug AT-527, a purine nucleotide prodrug, which has shown pan-genotypic efficacy against hepatitis C infection (Good et al., 2020) has also been considered against COVID-19 in a multinational clinical trial (Clinical trial no. NCT04396106). Apart from antiviral drugs, the strategies to tackle increased inflammatory responses during COVID-19 have also been investigated in various studies. Corticosteroids, due to their potent anti-inflammatory effects have gained importance in this regard. Numerous studies investigated a glucocorticoid-dexamethasone but its importance is recently highlighted in large scale RECOVERY trial (Horby et al., 2020a) and further gained recommendation of its use from various platforms. The daily dose of 6mg dexamethasone for 10days was used for hospitalized patients and showed reduced mortality on 28th day compared to the control groups (Horby et al., 2020a). Currently there are 45 registered clinical trials for corticosteroid use against COVID-19 (ClinicalTrials.gov, 2021b).

## Pharmacokinetics and Drug Interactions of Some Repurposed Drugs

Understanding the relationship between the pharmacokinetic properties and the therapeutic effect or side-effect of a drug is clinically important (Takahashi, 2000). The bioavailability, volume of distribution, protein binding, half-life, and elimination are the key determinants of successful drug therapy. Especially in severe COVID-19 cases, complex clinical situations may arise due to multiple organ failure and the consequences of drug action cannot be predicted without sufficient pharmacokinetic data (Zaim et al., 2020; Wang T. et al., 2020). The relevant information can be obtained from preclinical and large randomized clinical trials. However, clinicians will continue to confront the challenge of deciding the dosage of repurposed drugs until the pharmacokinetics parameters are better assessed in COVID-19. Furthermore, multi-drug therapy is unavoidable in the treatment of COVID‐19, especially for those patients with pre-existing diseases (Jafari et al., 2020). Therefore, drug-drug interactions (DDIs) are the major concern in clinical practice. It is too early to precisely estimate the effect of DDIs between the experimental drugs used to treat COVID-19 and other prescription drugs. Similarly, the impact of DDIs on pre-existing clinical conditions may not be clearly ruled out. Because, the currently available COVID-19 clinical results are mostly obtained from a relatively short-term study and was not performed in patients taking specific drugs for pre-existing illness (Sciaccaluga et al., 2020). Moreover, clinically significant DDIs can be rationalized in relevant studies performed on appropriate patient populations with high accuracy. Herein, we recapitulate the pharmacokinetics and DDIs of some COVID-19 repurposed drugs under consideration. Additionally, we report the *in silico* pharmacokinetics prediction of all repurposed drugs discussed in this review (Table 2).

**TABLE 2:** ADMET analysis of drugs repurposed against SARS-CoV-2.

Property	Model name	Predicted value										Unit
		**CQ**	**HCQ**	**AZM**	**RMD**	**RBV**	**LPV**	**RTV**	**FPV**	**UFV**	**OTV**	
Absorption	Lipophilicity	4.81	3.78	1.90	2.31	−3.01	4.32	5.90	−0.99	5.17	1.28	Numeric (LogP)
	Water solubility	−4.249	−3.627	−4.133	−3.07	−1.71	−4.819	−3.35	−2.12	−3.98	−2.47	Numeric (log mol/L)
	Caco2 permeability	1.62	1.54	−0.21	0.63	0.42	0.06	0.37	0.62	0.83	0.93	Numeric (log papp in 10^–6^cm/s)
	Intestinal absorption (human)	89.95	90.21	45.80	71.10	54.98	65.60	69.45	91.69	88.29	74.46	Numeric (% Absorbed)
	Skin permeability	−2.67	−2.84	−2.74	−2.73	−2.76	−2.73	−2.73	−3.2	−2.73	−3.17	Numeric (log Kp)
	P-glycoprotein substrate	Yes	Yes	Yes	Yes	No	Yes	Yes	No	Yes	No	Categorical (Yes/No)
	P-glycoprotein I inhibitor	No	No	Yes	Yes	No	Yes	Yes	No	Yes	No	Categorical (Yes/No)
	P-glycoprotein II inhibitor	No	No	No	No	No	Yes	Yes	No	Yes	No	Categorical (Yes/No)
Distribution	VDss (human)	1.33	1.07	−0.214	0.30	−0.01	−0.24	0.42	−0.21	0.72	0.04	Numeric (log L/kg)
	Fraction unbound (human)	0.19	0.24	0.51	0.005	0.78	0	0	0.78	0.12	0.59	Numeric (Fu)
	BBB permeability	0.349	0.07	−1.85	−2.05	−0.92	−0.83	−1.66	−0.12	0.03	−0.69	Numeric (log BB)
	CNS permeability	−2.19	−2.51	−3.77	−4.67	−3.75	−2.935	−3.29	−3.08	−2.19	−3.11	Numeric (log PS)
Metabolism	CYP2D6 substrate	Yes	Yes	No	No	No	No	No	No	No	No	Categorical (Yes/No)
	CYP3A4 substrate	Yes	Yes	Yes	Yes	No	Yes	Yes	No	Yes	No	Categorical (Yes/No)
	CYP1A2 inhibitior	No	Yes	No	No	No	No	No	No	No	No	Categorical (Yes/No)
	CYP2C19 inhibitior	No	No	No	No	No	Yes	No	No	Yes	No	Categorical (Yes/No)
	CYP2C9 inhibitior	No	No	No	No	No	Yes	Yes	No	No	No	Categorical (Yes/No)
	CYP2D6 inhibitior	Yes	Yes	No	No	No	No	No	No	Yes	No	Categorical (Yes/No)
	CYP3A4 inhibitior	No	No	No	No	No	Yes	Yes	No	Yes	No	Categorical (Yes/No)
Excretion	Total clearance	1.09	1.15	-0.42	0.19	0.62	0.45	0.56	0.51	0.68	0.92	Numeric (log ml/min/kg)
	Renal OCT2 substrate	Yes	No	No	No	No	No	No	No	No	No	Categorical (Yes/No)
Toxicity	AMES toxicity	Yes	Yes	No	No	No	No	No	No	No	No	Categorical (Yes/No)
	Max. Tolerated dose (human)	−0.16	−0.09	1.02	0.15	1.01	−0.29	0.09	1.29	0.33	0.47	Numeric (log mg/kg/day)
	hERG I inhibitor	No	No	No	No	No	No	No	No	No	No	Categorical (Yes/No)
	hERG II inhibitor	Yes	Yes	No	Yes	No	Yes	Yes	No	Yes	No	Categorical (Yes/No)
	Oral rat acute toxicity (LD50)	2.85	2.65	2.76	2.04	1.98	2.38	2.70	1.94	2.95	2.67	Numeric (mol/kg)
	Oral rat chronic toxicity (LOAEL)	1.02	1.40	1.99	1.63	3.09	5.94	2.23	2.02	0.73	1.09	Numeric (log mg/kg_bw/day)
	Hepatotoxicity	Yes	Yes	Yes	Yes	No	Yes	Yes	No	Yes	No	Categorical (Yes/No)
	Skin sensitization	No	No	No	No	No	No	No	No	No	No	Categorical (Yes/No)
	*T.pyriformis* toxicity	1.55	1.06	0.28	0.28	0.28	0.28	0.28	0.09	0.29	0.10	Numeric (log ug/L)
	Minnow toxicity	0.74	1.32	7.80	0.29	4.62	−1.50	1.787	3.40	−0.12	2.31	Numeric (log mM

The ADMET information about selected drugs is predicted using online server http://biosig.unimelb.edu.au/pkcsm/.

Generally, the drugs are evaluated for potential risk of DDIs during drug development stage to determine the effect of cytochrome P450 (CYP) and P-glycoprotein mediated interactions (Elmeliegy et al., 2020). However, a lack of published clinical data in this area is a major setback. Some efforts are made to document the potential DDIs and they can be accessed from the COVID-19 Drug Interactions site (Liverpool COVID-19 interactions, 2021) published by the Liverpool Drug Interaction Group and the IBM Micromedex Drug Interaction Checking site (IBM Micromedex, 2021) maintained by IBM Watson Health, Greenwood Village, Colorado, United States.

Two antimalarial drugs CQ and HCQ, with or without a macrolide antibiotic AZM, have been studied in multiple clinical trials for the treatment of COVID-19. QTc prolongation, Torsade de Pointes, ventricular arrhythmia, and cardiac deaths are major risks of CQ and HCQ. QT prolongation and potentially life-threatening arrhythmias with HCQ therapy originate from its pharmacodynamics action (O’Laughlin et al., 2016). CQ and HCQ are moderate inhibitors of cytochrome P450 (CYP) 2D6, and potential inhibitors of P-glycoprotein (P-gp) (Rendic and Guengerich, 2020). Therefore, these drugs cause a wide range of potential DDIs by altering the plasma concentration of several drugs. HCQ increases the plasma concentrations of amiodaron, dabigatran, edoxaban, cyclosporine, tacrolimus and sirolimus and decreases the bioavailability of carbamazepine and rifampicin with concomitant use (Liverpool COVID-19 interactions, 2021). The co-administration of HCQ with anti-tubercular drugs such as isoniazid or ethambutol increases the risk of peripheral neuropathy in diabetic patients. CQ and HCQ may decrease the activity of RDV and therefore co-administration of these drugs is not recommended. AZM is not metabolized by cytochromes P450 and it is not a substrate/inhibitor of CYP450. AZM is a known P-glycoprotein (P-gp) inhibitor and, if co-administered with P-gp substrates, it may result in increased serum levels requiring special therapeutic dose monitoring (Scherrmann et al., 2020).

RDV is a prodrug that inhibits viral RNA polymerases. The metabolic stability of RDV studied in various animal models showed that it was relatively stable in the intestine (t1/2 = 40.3–114.1min) but unstable in the liver (t1/2 < 3.9min) (FDA, 2020a). The hepatic instability and the complete first-pass effect prevented oral delivery of RDV. Therefore, the drug is administered through the intravenous route (IV). The IV administration of RDV (200mg) to healthy humans produced AUC0-24 values of 4.8μM/h with moderate protein binding. The *in vitro* metabolism studies of RDV suggest that it was predominantly metabolized by CYP2C8, CYP2D6, and CYP3A4. It is extensively metabolized in hepatic tissues, and the rate of metabolism by CYP3A4 alone was estimated as 42.1%. The elimination studies carried out in rats and monkeys showed that kidney and bile excretion were the major routes of elimination of RDV. It has a low potential for significant drug-drug interactions because of its rapid clearance. However, the antiviral activity effect of RDV is reduced when co-administered with CQ or HCQ (COVID-19 treatment update, FDA). It is because of the interference of CQ on the intracellular metabolic activation of RDV. Therefore, the co-administration of inhibitors of such CYPs can lead to a potentially high risk of toxic effect (Cattaneo et al., 2020). In a case study it was reported that RDV induced acute hepatotoxic effect in a male COVID-19 patient and realized the toxic effect was due to probable interaction of P-glycoprotein (P-gp) inhibitors (Leegwater et al., 2020). The clinical history of the patient described that the patient was treated with the P-gp inhibitors like chloroquine and amiodarone along with RDV. An adverse effect of an increase in hepatic transaminase activity was also observed in the clinical trial of RDV. RDV was not genotoxic, and it does not impair male fertility (Singh et al., 2020). Based on these preliminary findings, the FDA had granted a EUA for RDV for the treatment of COVID-19 patients (Ison et al., 2020; FDA, 2020d) and was last reissued on October 22, 2020 with some amendments.

The combination of LPV and RTV was approved for the treatment of HIV infection and has recently been investigated in COVID-19 patients (Jean et al., 2020). RTV-boosted LPV (400/100mg) was orally administered to COVID-19 patients. LPV is predominantly metabolized by CYP3A4 isoenzyme, and RTV is a strong inhibitor of CYP3A4 (Chen, 2005; Gregoire et al., 2020). Therefore, RTV prevented the metabolism of LPV. The concentrations of LPV in COVID-19 patients were extremely high compared with HIV-infected patients. No severe adverse events were reported in the clinical trials of LPV and RTV. However, these two drugs can inhibit metabolism and increase plasma levels of several drugs that may induce toxic effects. The potentially severe DDIs were recorded for the concurrent administration of HCQ and LPV/RTV in hospitalized COVID-19 patients (Cattaneo et al., 2020). Cattaneo, *et al.*, reported that more than fifty percent of category D based DDIs and they are attributed to LPV/RTV. The risk of QT interval prolongation by LPV/RTV therapy may be due to inhibition of human ether-a-go-go related gene (hERG) (Sciaccaluga et al., 2020). The cardiotoxicity risk ratio of LPV/RTV is double that of HCQ and AZM (Cattaneo et al., 2020). Furthermore, RTV is shown to increase in the bioavailability and half-life of immunosuppressant drugs such as tacrolimus and cyclosporine by inhibition of CYP3A (Zijp et al., 2020).

The clinical trial results of FPV showed that the peak plasma concentration was achieved at 2h after oral administration (Du and Chen, 2020). The plasma protein binding of FPV was observed 54% in humans. FPV is metabolized in the hepatic tissues majorly by aldehyde oxidase (AO), and partly by xanthine oxidase (Gowen et al., 2015). The metabolites of FPV are rapidly excreted by the kidneys. Particularly, FPV is a mechanism-based AO inhibitor and affects the action of AO in a concentration‐dependent manner. Furthermore, potential DDIs between FPV, cimetidine, and zaleplon have been already reported (Renwick et al., 2002). The chances of occurring DDIs between FPV and citalopram, famciclovir, zaleplon and sulindac are higher as these drugs are also metabolized by AO (Du and Chen, 2020). *In vivo* study showed inhibitory effect of FPV on CYP2C8 isoenzyme. Therefore, more caution was necessary with anticancer agents such as tamoxifen (AO inhibitor) and paclitaxel (CYP2C8 substrate) (Jafari et al., 2020). Furthermore, a clinical study showed that FPV increases the concentrations of antidiabetic drugs such as pioglitazone or repaglinide with concomitant use that leads to the risk of hypoglycemia. Therefore, a great deal of attention must be paid by clinicians in designing the therapeutic dosage regimen.

## Conclusion

For containing the devastating scenario of COVID-19 pandemic, the identification of potent and less toxic therapeutics for COVID-19 is a key research priority. Current research efforts are intensified on the evaluation of existing drugs against SARS-CoV-2 infection. Despite several challenges in SARS-CoV-2 infection, the drug repurposing strategy has proved its important role in the rapid discovery of an effective treatment for COVID-19. Only judicious evaluation of these repurposed drugs may show real insights on its clinical effectiveness and clinical safety in COVID-19 patients. Furthermore, it is essential to address the issue of drug-drug interaction of the repurposed drugs in COVID-19 patients with comorbidities (Jakhmola et al., 2020c). In our knowledge, the present review adequately provides all relevant information currently needed to assist clinicians and researchers working in this area.
